# Assessment in undergraduate medical education: a review of course exams

**DOI:** 10.3402/meo.v18i0.20438

**Published:** 2013-03-06

**Authors:** Allison A. Vanderbilt, Moshe Feldman, Isaac K. Wood

**Affiliations:** 1 Center on Health Disparities, School of Medicine, Virginia Commonwealth University, Richmond, VA, USA; 2 Office of Assessment and Evaluation Studies, Center for Human Simulation and Patient Safety, School of Medicine, Virginia Commonwealth University, Richmond, VA, USA; 3 Medical Education and Student Affairs, School of Medicine, Virginia Commonwealth University, Richmond, VA, USA

**Keywords:** undergraduate medical education, assessment, course exams, NBME

## Abstract

**Introduction:**

The purpose of this study is to describe an approach for evaluating assessments used in the first 2 years of medical school and report the results of applying this method to current first and second year medical student examinations.

**Methods:**

Three faculty members coded all exam questions administered during the first 2 years of medical school. The reviewers discussed and compared the coded exam questions. During the bi-monthly meetings, all differences in coding were resolved with consensus as the final criterion. We applied Moore's framework to assist the review process and to align it with National Board of Medical Examiners (NBME) standards.

**Results:**

The first and second year medical school examinations had 0% of competence level questions. The majority, more than 50% of test questions, were at the NBME recall level.

**Conclusion:**

It is essential that multiple-choice questions (MCQs) test the attitudes, skills, knowledge, and competency in medical school. Based on our findings, it is evident that our exams need to be improved to better prepare our medical students for successful completion of NBME step exams.

Adequately measuring core competencies such as medical knowledge is an essential component for evaluation, the provision of reliable feedback, and improving medical education (1, 2). Summative assessment methods used to evaluate medical knowledge have predominantly included multiple-choice questions (MCQs) assessing recall of medical knowledge or facts. This highlights the need to more rigorously evaluate medical students by measuring outcomes that reflect higher order processes such as the application of knowledge (3). Professional oversight bodies such as National Board of Medical Examiners (NBME), American Association of Medical Colleges (AAMC), and the Liaison Committee on Medical Education (LCME) are challenging medical schools to undertake curricular reforms, including assessment procedures that will result in medical students exercising and developing critical thinking skills from the time of matriculation through their life-long practice of medicine (4–6).

MCQs have been widely used for summative assessment in undergraduate medical education because of convenient standardization, efficient testing for large classes, and broad sampling of knowledge (7, 8). A major disadvantage of MCQs is they are often poorly written in a way that test memory recall of independent facts rather than application of knowledge (7). A well-constructed MCQs can test higher order diagnostic thinking and application of knowledge, evaluating the examinee's ability to integrate, synthesize, and judge medical information (3, 7, 9). However, writing MCQs that evaluate application of knowledge can be challenging, and most faculties are not formally trained (10); therefore, making it difficult to develop test questions that require application and critical thinking skills. This lack of training results in the development of basic recall MCQs administered to students to assess their learning, whereas faculty should develop application MCQs that require students to think through a series of steps and use their knowledge/learning in order to properly answer the questions. The NBME has defined a recall question as one that assesses examinee knowledge and definitions or isolated facts (3). Application questions have been defined as requiring an examinee to reach a conclusion, make a prediction, or select a course of action (3).

The purpose of this study is to describe an approach for evaluating assessments implemented during the first 2 years of medical school and report the results of applying this method to current first and second year medical student examinations at Virginal Commonwealth University (VCU) School of Medicine (SOM). We reflect on our findings and recommend that medical schools should review their current exam questions to assure that they are sufficiently assessing and preparing their students for the NBME board examinations. There have been several studies that have used Bloom's taxonomy to address assessment in medical schools (11–13). However, we chose the Moore's framework (14) approach to address our exam questions to determine if we are in alignment with the NBME definitions for recall and application reflected in Moore's Level 3a and 3b. This review of exams was to determine the level that our learners are performing at in medical school so that we can develop high quality exam questions that will measure knowledge as defined by the NBME.

## Methods

**Fig. 1:**
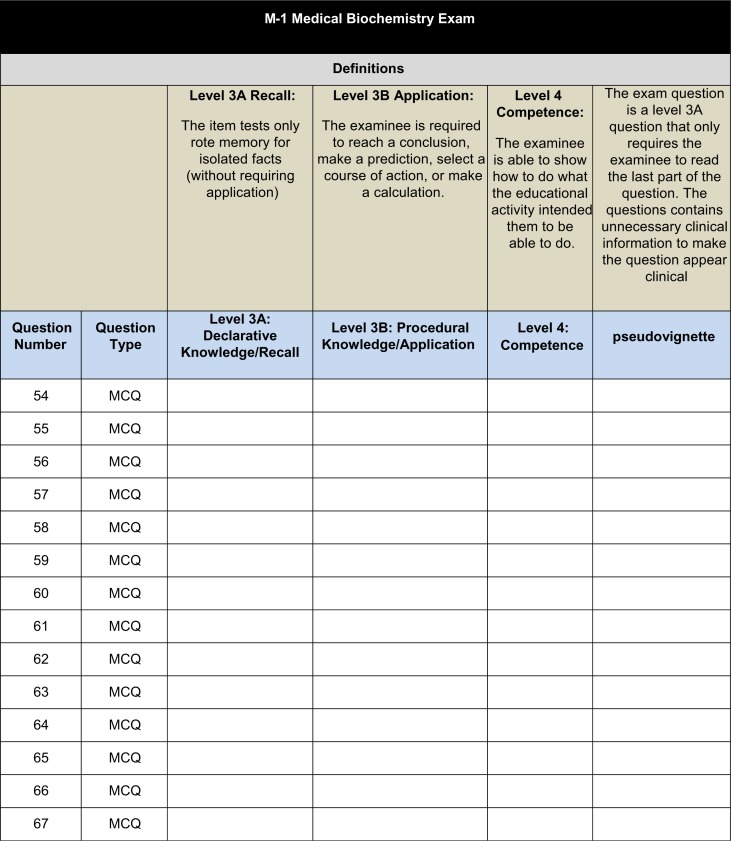
Example of coding form.

Three faculty members coded all exam questions administered during the first 2 years of medical school. Each faculty member was provided with a hard copy of each exam and an excel spreadsheet (coding sheet) that corresponded to each individual exam (Fig. 1). The three reviewers coded each exam question independently to determine the “type” of question that was asked; more specifically, to determine if the exam question was a recall, application, or competence question.

The theoretical framework that we applied to our coding of exam questions was based on Moore's framework (14). Moore's expanded outcomes framework (Table 1) was selected to organize and categorize all test questions for first and second year medical school examinations. Moore's framework accounts for outcomes representing learners’ participation (Level 1), satisfaction (Level 2), and knowledge (Level 3), and it is sensitive to higher level changes demonstrated as competence (Level 4), performance (Level 5), patient health (Level 6), and community health outcomes (Level 7). This framework was selected because it allows for assessment of learners at higher levels beyond competence. We selected this model over the NBME recall and application to determine if we are able to develop assessments for our medical students at higher levels to further develop their critical thinking skills. Moore's framework aligns nicely with NBME and allows for the matching definitions for recall and application; to apply at Level 3 related to knowledge of learning. Furthermore, with the Moore's framework we had the potential to measure our students’ learning at the performance level with potential implications at a patient health level. We expected the exam questions to fall into one of three categories, Level 3a, 3b, or 4.

**Table 1:** Moore's Expanded Outcomes Framework

Outcomes framework	Description	Sources of data
Participation Level 1	Number of learners who participate in the educational activity	Attendance records
Satisfaction Level 2	Degree to which expectations of participants were met regarding the setting and delivery of the educational activity	Questionnaires/surveys completed by attendees after an educational activity
Learning: declarative knowledge Level 3a	The degree to which participants state *what* the educational activity intended them to know	Objective: pre- and post-tests of knowledge Subjective: Self-report of knowledge gain
Learning: procedural knowledge Level 3b	The degree to which participants state *how* to do what the educational activity intended them to know how to do	Objective: pre- and post-tests of knowledge Subjective: self-reported gain in knowledge (e.g. reflective journal)
Competence Level 4	The degree to which participants *show* in an educational setting *how* to do what the educational activity intended them to be able to do	Objective: observation in educational setting (e.g. online peer assessment and EHR chart stimulated recall.) Subjective: self-report of competence; intention to change
Performance Level 5	The degree to which participants *do* what the educational activity intended them to be able to do in their practices	Objective: observed performance in clinical setting; patient charts; administrative databases Subjective: self-report of performance
Patient health Level 6	The degree to which the health status of patients improves due to changes in the practice behavior of participants	Objective: health status measures recorded in patient charts or administrative databases Subjective: patient self-report of health status
Community health Level 7	The degree to which the health status of a community of patients changes due to changes in the practice behavior of participants	Objective: Epidemiological data and reports Subjective: Community self-report

Upon completion of individually coding each exam, the reviewers met bi-monthly. The reviewers discussed and compared the coded exam questions. During the bi-monthly meetings all differences in coding were resolved with consensus as the final criterion. Prior to the bi-monthly meetings, the lead author and a graduate assistant would go through all three coded sheets to identify the discrepancies among the reviewers. The exam questions that were not in agreement were then tagged/marked for discussion. The three researchers then would meet and discuss each item that was not in agreement. Each individual would explain why he or she selected the code (recall or application) for the item and consensus would be met. Each meeting was scheduled for a 2-h interval and the research team met bi-monthly for over the course of a 10-month time period. One investigator kept the master copy of the final decision.

The current curriculum in the SOM at VCU is based on a two by two curriculum. The first 2 years are basic science (pre-clinical years) and the third and fourth years are focused on the standard clinical experiences required by LCME. Therefore, with the changes coming with our new integrated curriculum, it was necessary for us to evaluate our current examinations to determine what if any exams need to be revised/improved based on the Moore's framework in alignment with the NBME.

### Data analysis

All data were analyzed in SPSS 18.0. Frequency data were calculated to determine the percentage of exam questions that were declarative knowledge (Level 3a), procedural knowledge (Level 3b), or competence (Level 4).

## Results

### First year medical school examinations

There were 22 examinations administered and reviewed during the first year of medical school. These exams included the following basic science content areas: biochemistry, population medicine, history, physiology, immunology, gross and development anatomy, medical biochemistry, human genetics, and practical exams focused on history. Fourteen percent (n=3) of the examinations administered had more than 50% of the test questions that were coded as procedural knowledge (application). Eighty-six percent (n=19) of the examinations administered had more than 50% of the test questions that were coded as declarative knowledge (recall). The first year medical school examinations had 0% of competence questions.

### Second year medical school examinations

There were 23 examinations administered during the second year of medical school that were reviewed. They included the following basic science content areas: behavioral sciences, neurosciences, hematology–oncology, microbiology, endocrine pathology, cardiovascular, Gastroinestinal, musculoskeletal, pathogenesis, pharmacology, renal, respiratory, nervous system, and women's health. Eighty-five percent (n=17) of the examinations administered had more than 50% of the test questions that were coded as procedural knowledge (application). Fifteen percent (n=6) of the examinations administered had more than 50% of the test questions that were coded as declarative knowledge (recall). The second year medical school examinations had 0% of competence questions.

## Discussion

High quality questions are important for medical student assessments (7). This is even more essential for summative assessments where medical student performance influences their progression within the program (7) and results in a final grade for a course. Problem-solving questions involving short vignettes can be used to assess application of knowledge (procedural knowledge) rather than simple factual recall (declarative knowledge) (8). Such questions can be used to test basic as well as clinical sciences. Writing such questions requires training and experience, and is therefore costly.

If the vignettes are not constructed correctly, the examination question can become a pseudovignette. A pseudovignette is a “clinical” vignette that ends with a declarative knowledge (recall) question. Therefore, all of the information provided in the vignette is not necessary to answer the question. Pseudovignettes can be prevalent within an examination that is written by a faculty member who has not received training or support in test development. It is time consuming and challenging to write a true clinical vignette to ask basic science questions; however, this can lead to better test questions and the assessment of student knowledge at a higher level.

Based on these findings at VCU SOM we are going to develop a workshop. This workshop will be provided to the first and second year Course Directors on question writing using Moore's framework. Faculty will be provided with a template for preparing questions. As questions are developed, a team of individuals who have experience in writing NBME-like questions will review the bank using the Moore rating scale. If a question is declarative, the committee will suggest revisions to make the question procedural. For example, developing a multiple-choice question that aligns with both the Moore's framework of Level 3b as well as the application definition from NBME. This will occur through the revising exam questions through the support of text writing experts located at VCU SOM. This will be reviewed by the Course Directors to ensure that the revision does not alter the proposed content. The committee will also decide if the question addresses more than one domain (e.g., hematology/oncology and ethics) and it will make these assignments. The questions will be placed in a bank that may be used for forming new examinations. We believe these steps will improve the psychometric support needed to develop higher quality assessments for the medical students at VCU SOM.

### Limitations

We acknowledge that our study is limited to the exams that are administered during the first and second year of medical school at VCU SOM. The findings and conclusions that we draw are based solely on findings specific to the examination process at VCU SOM and it will be difficult to make generalizations about exams to other medical schools. However, medical schools can follow our method to guarantee that their medical school examinations are assessing their students at the procedural knowledge level rather than the declarative knowledge level.

## Conclusion

It is essential that MCQs test the attitudes, skills, knowledge, and competency in medical school. Based on our findings, it is evident that our exams need to be improved to better prepare our medical students for successful completion of NBME step exams. Therefore, we are developing and preparing in-house faculty experts to assist with the development and maintenance of high quality (application, competence, and beyond) exam questions. We highly recommend that medical schools take the time and effort to evaluate their current assessments to ensure that students are being asked the application questions necessary to measure student learning.
